# Reservoir‐Type Subcutaneous Implantable Devices Containing Porous Rate Controlling Membranes for Sustained Delivery of Risperidone

**DOI:** 10.1002/adhm.202403689

**Published:** 2025-01-16

**Authors:** Linlin Li, Andi Dian Permana, Juan Domínguez‐Robles, Muh Nur Amir, Habibie Habibie, Qonita Kurnia Anjani, Li Zhao, Natalia Moreno‐Castellanos, Ryan F Donnelly, Eneko Larrañeta

**Affiliations:** ^1^ School of Pharmacy Queen's University Belfast Lisburn Road 97 Belfast BT9 7BL UK; ^2^ Faculty of Pharmacy Hasanuddin University Makassar 90245 Indonesia; ^3^ Department of Pharmacy and Pharmaceutical Technology Faculty of Pharmacy University of Seville Seville 41012 Spain; ^4^ CICTA Department of Basic Sciences Medicine School Health Faculty Universidad Industrial de Santander Cra 27 calle 9 Bucaramanga 680002 Colombia

**Keywords:** Implantable devices, poly(caprolactone), porous membranes, risperidone, sustained delivery

## Abstract

Implantable drug delivery systems are crucial for achieving sustained delivery of active compounds to specific sites or systemic circulation. In this study, a novel reservoir‐type implant combining a biodegradable rate‐controlling membrane with a drug‐containing core prepared using direct compression techniques is developed. The membrane is composed of poly(caprolactone) (PCL), and risperidone (RIS) served as the model drug. Characterization of both membranes and direct compressed pellets includes hardness testing, optical coherence tomography, mercury intrusion porosimetry, and surface morphology observation. In vitro release studies of RIS reveal that higher drug loading in the pellets extended‐release duration up to 70 days when incorporated into membranes with four layers. Increasing the number of membrane layers slows the release rate further, ranging from 70 to 170 days depending on membrane thickness. Biocompatibility studies demonstrate that these implantable devices are non‐toxic and biocompatible with cells in vitro. In vivo studies conduct in male Wistar rats demonstrate sustained release of RIS, with plasma levels showing a significant increase post‐implantation at a relatively constant rate for up to 49 days. These results indicate that the developed implants have the potential to provide long‐acting drug delivery to the systemic circulation.

## Graphical abstract

This study presents a novel implantable drug delivery systemwith a biodegradable poly(caprolactone) membrane and compressed drug core forsustained release. These implantable devices deliver risperidone in vitro for 170 days and in vivo for 49 days in rats, showing promise for chronic conditions like schizophrenia.

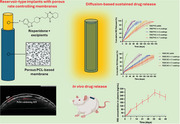

## 1 Introduction

Implantable drug delivery systems (IDDS) are typically medical devices designed to deliver drugs to specific sites of action or systemic circulation in a sustained way.^[^ ^1^ ^]^ Deansby and Parkes first applied this idea of IDDS in practice in 1938 when they implanted compressed pellets of crystalline estrone subcutaneously to investigate their influence in animal models.^[^ ^2^ ^]^ IDDS, as a long‐acting delivery strategy, offers many advantages. It allows targeted and localized drug delivery and can also bypass first‐pass metabolism and degradation in the stomach compared to oral delivery systems. This leads to faster drug release and lower concentrations of drug required to achieve therapeutic effect. Therefore, it might potentially reduce side effects and improve patient compliance.^[^ ^3^ ^]^ Although surgical procedures are required to initiate therapy, the long‐acting delivery ranging from several weeks to months, or even a year, may outweigh this disadvantage.^[^ ^3^ ^]^

It is difficult to give a classification of IDDS because of several exceptions and hybrids which may belong to multiple categories. Biodegradable implants have more advantages than other categories because the polymers used are biodegradable and biocompatible, which guarantees safe absorption or excretion from the body. Moreover, the surgical procedure to remove the implant is not required, which can also potentially improve patient compliance. Most biodegradable implantable devices are made from a mixture of polymer and drug. After implantation, the drug will be released with the degradation of the polymer.^[^ ^4^ ^]^ The release rate can be affected by different factors, such as the degradation rate of different polymers in vivo, the drug loading, and the solubility and permeability of the drug.^[^ ^4^ ^]^ By changing these factors, the implant can be designed with different predetermined release rates. The polymers used to fabricate biodegradable implantable devices are mostly thermoplastic aliphatic polyesters such as poly(lactic acid) (PLA), poly(glycolic acid) (PGA), poly(lactic*‐co*‐glycolic acid) (PLGA), and poly(caprolactone) (PCL). Ester, amide, and anhydride bonds are labile bonds that can be hydrolyzed or degraded by enzymes contained in these polymers. These polymers have been extensively researched due to their advantageous traits including biodegradability, biocompatibility, and mechanical strength.^[^ ^5^ ^]^

PCL has been one of the most commonly used biodegradable polymers in IDDS because of its biocompatibility, biodegradability, lack of toxicity, and comparatively inexpensive price.^[^ ^6^ ^]^ It has become an FDA‐approved polymer that can be used in the fabrication of medical devices.^[^ ^7^ ^]^ For example, it has been used in a long‐term contraceptive device Capronor containing levonorgestrel.^[^ ^8^ ^]^ The degradation time of PCL is longer than other polymers such as PLA, PGA, and PLGA, ranging from several months to years. The degraded products can either be metabolized in the tricarboxylic acid cycle or excreted through the kidney.^[^ ^9^ ^]^

A potential use of biodegradable IDDS is the treatment of chronic mental illness as a sustained pharmacological treatment is needed. There are many injectable formulations that provide sustained drug delivery for schizophrenia treatment.^[^ ^10^ ^]^ However, the use of solid implants could be used to extend the duration of the treatment. To date, a subcutaneous implant named DLP‐114 used to treat schizophrenia is in phase II clinical trials.^[^ ^11^ ^]^ This IDDS is developed by Delpor (Delpor Inc., San Francisco, CA, USA) using ProzorTM technology.^[^ ^12^ ^]^ A small cylindrical reservoir with membranes at both ends is used as a carrier to load risperidone (RIS), an antipsychotic drug, and some excipients, which can maintain the acidic environment and then improve the solubility of the drug.^[^ ^12^ ^]^ The Phase I trials have shown that it can maintain constant plasma levels with little fluctuation for several months and distribute the medicine in a zero‐order manner.^[^ ^11^ ^]^ Meanwhile, a subcutaneous RIS implant was developed and applied in adult patients with schizophrenia to assess its pharmacokinetics. This RIS implant achieved therapeutic levels within around 2 days after implantation and released for 6 months at a constant rate. Phase II and III studies are currently being conducted to assess the safety and efficacy of RIS implants.^[^ ^13^ ^]^

In this study a simple method to develop reservoir‐type implants combining a biodegradable rate‐controlling membrane and a drug‐containing core prepared by direct compression of pharma excipients and RIS. Direct compression is the method using compression to produce solid dosage forms from the blend of ingredients, which has advantages such as low cost and simple operation.^[^ ^14^ ^]^ The aim of this work was to design and develop RIS implantable devices for use with PCL. PCL films and directly compressed RIS pellets were fabricated separately and then incorporated. Following these steps, a series of characterizations, in vitro release studies, and in vitro biocompatibility studies were performed to select the most appropriate formulation, which can provide sustained drug release and then be applied to the animal study.

## 2 Results and Discussion

### 2.1 Fabrication and Characterization of Directly Compressed RIS Pellets

Direct compression is a manufacturing process that produces solid dosage forms from a mixture of active ingredients and excipients.^[^ ^14^ ^]^ Due to the advantages of low cost, shorter processing time, and simple operation, as well as the absence of the need for heat and/or solvents, it is preferred as the fabrication method for tablets or pellets.^[^ ^15^ ^]^ Besides, it is the method commonly used in the pharmaceutical industry, thus scaling it up will be simple.^[^ ^16^ ^]^

In this case, a small punch and die system (3 mm) was used to prepare the directly compressed pellets using a laboratory hydraulic press and a force of 1 ton applied for 20 s. These conditions are like the ones described previously for directly compressed tablet manufacturing.

As detailed in **Table** **1**, formulation R100 could not be formed, as the 100% RIS composition of the formulation adhered to the die and could not be removed. For well‐formed and consistently directly compressed pellet preparation, the use of suitable excipients is vital. In this case, poly(ethyelene glycol), with a molecular weight of 8000 Da (PEG 8000), and Hydroxypropyl β‐cyclodextrin (HP‐βCD) were used. PEG 8000, plays several vital roles in pharmaceutical formulations including solubilizing agent, matrix former, lubricant, plasticizer, and vehicle for drug delivery, due to its biocompatibility, non‐toxicity, and regulatory acceptance.^[^ ^17^ ^]^ HP‐βCD, a cyclic oligosaccharide derivative, is also widely used as an excipient in pharmaceutical formulations. Some of the functions of HP‐βCD as an excipient include a solubilizing agent, stabilizer, permeation enhancer, and compatibility agent.^[^ ^18^ ^]^ As shown in Table 1, five types of pellets have been fabricated, and most of the resultant pellets were robust under visual inspection. In this case, formulations R40P60, R50P50, R60P40, and R60C40 were selected to evaluate the influence of drug loading and the type of excipient in the drug release kinetics.

**Table 1:** Summary of appearance of directly compressed pellets containing RIS.

Formulation	Morphology	Observation
R40P60	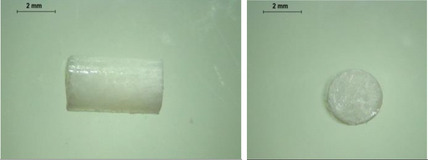	Well formed Robust
R50P50	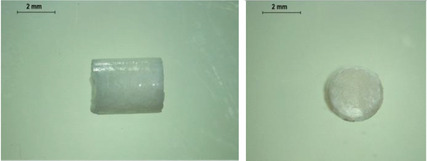	Well formed Robust
R60P40	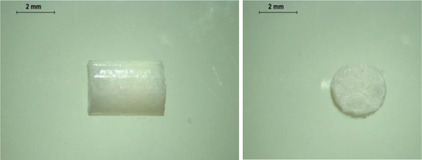	Well formed Robust
R75P25	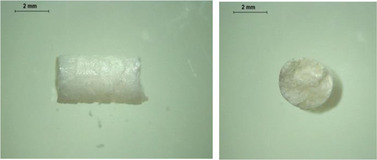	Formed with a rough surface Part of the formulation sticked to the die, made it hard to remove intact
R100	N/A	Did not form well the formulation sticked to the die, made it hard to remove intact
R60C40	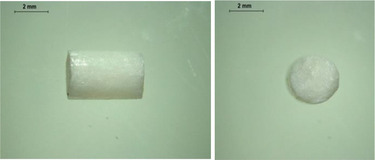	Well formed Robust

Directly compressed formulations have been evaluated before to prepare implants for sustained drug release.^[^ ^19^ ^]^ In these studies, the excipients selected to prepare the directly compressed pellets were NaCl and poly(vinyl pyrrolidone). In these studies, the method proposed was similar to the one developed here. Drugs were combined with excipients and subsequently compressed. Alternatively, other authors tried a combined approach that requires the combination of the drug with a thermoplastic, such as poly(lactide*‐co*‐glycolide), via compression/thermal processing.^[^ ^20^ ^]^ The direct compression method used in the present work is scalable and does not require the use of temperature that could lead to the degradation of the drug cargo during manufacturing.

The hardness of the resulting pellets was evaluated. The purpose of testing the hardness of pellets is to evaluate the maximum force needed to fracture a pellet. Hardness plays an important role in further assembly processes and in transit. According to the results listed in **Table** **2**., all pellets with different compositions performed well, with no significant difference in hardness (*p* > 0.05) observed. These hardness results are in line with previously reported directly compressed pellets prepared with pharmaceutical excipients such as microcrystalline cellulose.^[^ ^21^ ^]^ Accordingly, the obtained pellets could be handled appropriately. Higher hardness values will make the pellet disaggregation extremely slow impacting the amount of drug releases from the implants.^[^ ^22^ ^]^ This is an important parameter as there is a balance between release duration and the amount of drug release to achieve therapeutic outcomes.

**Table 2:** Response values for pellets containing RIS. (means ± s.d., *n* = 5).

Formulation	Hardness [N]
R40P60	38.2 ± 9.31
R50P50	30.6 ± 6.35
R60P40	28.8 ± 8.67
R60C40	30.8 ± 6.42

### 2.2 Fabrication of PCL Films

**Figure 1:**
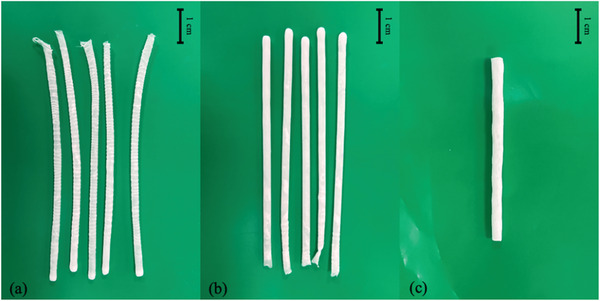
Physical appearance of films with different concentrations of PCL. a) 20% PCL in DCM b) 30% PCL in DCM c) 40% PCL in DCM.

PCL‐based membranes were prepared by coating a rotary metal rod using PCL solutions in dichloromethane (DCM) as described in the Experimental Section. Several cycles of casting were required to achieve suitable membrane thickness. To determine a suitable composition and concentration of PCL film, three different concentrations of PCL solution in DCM were used to fabricate the PCL films. As shown in **Figure** **1**a, films fabricated with a 20% PCL solution were formed. It is worth noting that due to the thin thickness of the film, it is not sufficiently hard to resist the force needed to remove the film from the rod, resulting in a rough surface. This may potentially affect the integrity of the films. Figure 1b shows the films fabricated with a 30% PCL solution. A smooth surface and uniform thickness can be observed. Films made from a 40% PCL solution are shown in Figure 1c. The higher concentration led to a higher viscosity in this case, therefore they did not spread evenly across the surface of the metal rod, resulting in a rough and nonuniform surface of the films.

**Figure 2:**
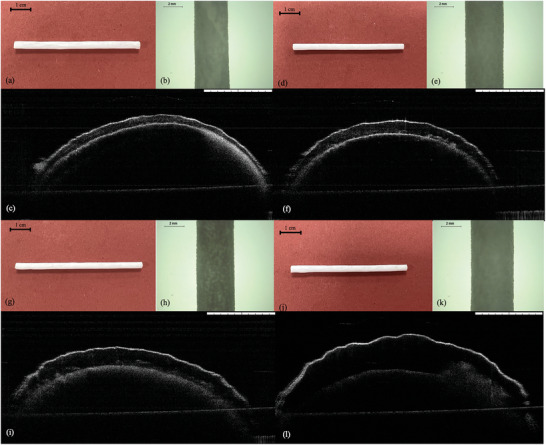
Physical appearance and OCT images of cross‐section of 30% PCL films with different thicknesses. a–c) 4 coatings d–f) 5 coatings g–i) 6 coatings j–l) 7 coatings. The scale bar of OCT images is 1 mm.

The 30% PCL formulation was chosen to fabricate films with different thicknesses of coatings. The physical appearance of the films is shown in **Figure** **2** All the films were formed well, with proper thickness and smooth and uniform surface.

Similar methods have been used before to obtain tubular membranes/scaffolds. These methods normally use electrospinning or 3D‐printing on the surface of a rod or a cylindrical structure to obtain tubular objects.^[^ ^23^ ^]^ An alternative to this is the use of hot‐melt extrusion but this technique normally requires the use of large amounts of polymer and therefore it is not ideal for the preparation of small prototypes.^[^ ^24^ ^]^

**Figure 3:**
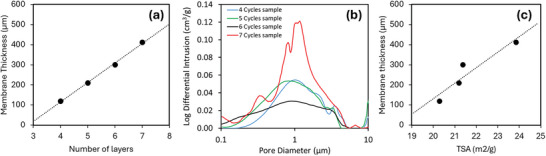
Thickness of membranes with different number of layers (a) (means ± s.d., *n* = 5). Pore size distribution curves of membrane samples (b). Correlation between total surface area (TSA) and membrane thickness (c).

Optical coherence tomography (OCT) is an imaging technology that evaluates biological tissue. However, it can be used to visualize other samples including pharmaceutical products or medical devices.^[^ ^25^ ^]^ In this case, OCT is used to observe the cross‐section of films and assembled implantable devices. Figure 2a,d,g,j shows the images of films with different layers of coatings, in which it can be seen that every film is uniform, and no gap can be observed. Different thicknesses can also be observed, as shown in **Figure** **3**a; the thickness increased with the increase of layers of coatings in a linear way. The thickness of the membranes for 4, 5, 6, and 7 layers was: 120 ± 9, 211 ± 6, 300.67 ± 8, and 412 ± 10 µm respectively. The coating process was consistent, with a significant difference (*p* < 0.05) observed in these four films with different layers of coatings. These results indicate that the method proposed can be easily adapted to modify membrane thickness and that the overall thickness of the membrane will be simple to predict as there is a linear relationship between the number of layers and the membrane thickness. The thickness of these membranes is in line with previously reported films used as rate‐controlling membranes in implantable devices ranging between 70 and 400 µm.^[^ ^19^, ^24^, ^26^ ^]^

The porosity of the membranes was evaluated using MIP (Figure 3b). All the analyzed samples had a similar monomodal pore size distribution with a peak value centered around 1 µm. However, the addition of more layers in the 7 coatings membranes sample produced the most uniform shape with an important increase of the pore size distribution intensity, which indicates a larger number of pores with same size of around 1 µm. Therefore, the thickness increase of the 7 coatings membrane samples resulted in a larger number of pores of the same size (around 1 µm) than the rest of the membrane samples. The obtained pore distribution is in line with previously reported PCL‐based membranes showing pores ranging between 1 and 2 µm.

The outcomes shown in **Table** **3** evidenced an increase of the membrane total surface area (TSA) associated to the general increase of pores population after increasing the number of coatings. These results also confirmed the general trend by which most of the membranes had higher values of the total pore volume (TPV) when the number of coatings was increased as can be seen in Figure 3c. Finally, the higher pore in volume is directly correlated with a higher porosity percentage, as reported by Stewart et al.^[^ ^27^ ^]^ It is important to mention that this is a more consistent method for preparing rate‐controlling membranes for implantable devices. Previous studies reported the use of this type of membrane, but they were wrapped around a drug‐containing core and sealed.^[^ ^28^ ^]^ This procedure is more time‐consuming and requires extensive membrane sealing in the edges to avoid drug leakages. This is minimized if membranes are prepared in a tubular shape.

**Table 3:** Textural properties of the polymeric membranes obtained by MIP.

Sample	TSA [m^2^ g^−1^]	TPV [cm^3^ g^−1^]
**4 coatings**	20.281	0.089
**5 coatings**	21.195	0.108
**6 coatings**	21.374	0.089
**7 coatings**	23.846	0.138

**Figure 4:**
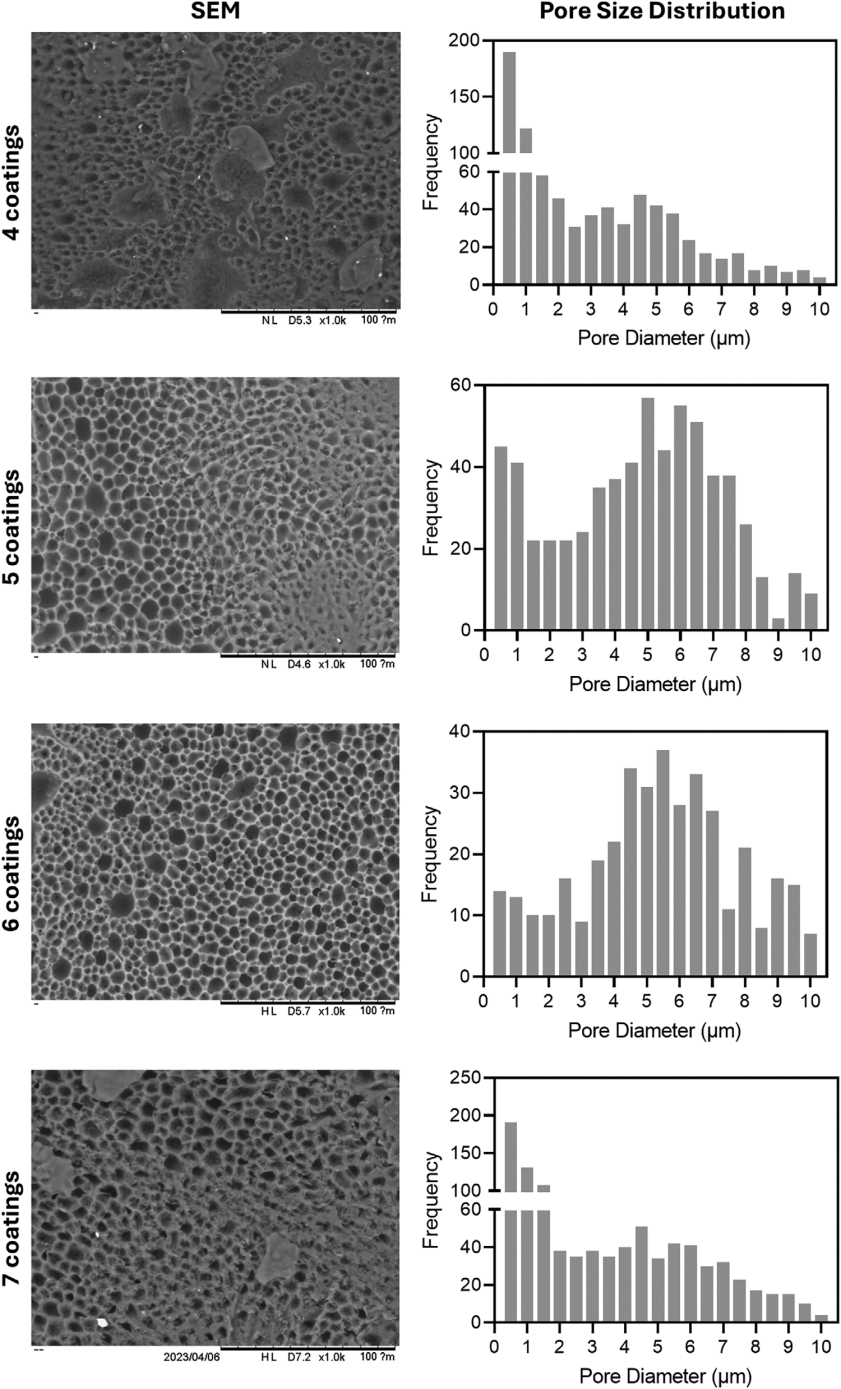
SEM images of the membranes and pore size distribution obtained from SEM images. Scale bar: 100 µm.

**Figure** **4** shows SEM images of the membranes with different coatings. The membranes exhibit pore sizes ranging between 2 and 8 µm in diameter. These results are consistent with the pore sizes obtained using MIP (Figure 3b). It is important to note that for some membranes, multiple small pores slightly distort the pore distribution. The pore size distribution is represented as the frequency of the pores. These small pores constitute a small portion of the total pore area. The results from MIP indicate that these small pores do not contribute to the differential intrusion (Figure 3b). Additionally, the frequency of pores ranging between 2 and 8 µm is similar across all samples, regardless of the number of coatings.

### 2.3 Preparation of RIS Implantable Devices and In Vitro Drug Release Experiments

**Figure 5:**
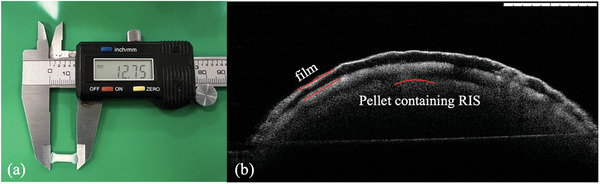
a) Image of an accomplished RIS implantable device. b) Representative OCT image of the cross‐section of the implantable device with an R60P40 formulated pellet in 4 coatings film. The scale bar of OCT images is 1 mm.

The mini RIS implantable device was assembled from a RIS pellet and a PCL tube. The melting point of PCL is between 50 and 60 °C,^[^ ^29^ ^]^ so the sealing process can be easily performed using heated pliers. By adjusting the number of pellets loaded into the PCL tube, the drug loading of this implantable device can be adjusted. **Figure** **5**a presents a completed RIS implantable device. The OCT cross‐section image of an implantable device is shown in Figure 5b. In this case, it shows the RIS pellet was right filled in the film. The outer part is clearer than the inner part due to the penetration of the laser.

**Figure 6:**
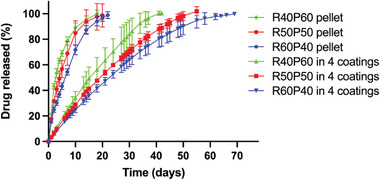
In vitro cumulative release profile of PEG‐RIS pellets. Release studies were carried out in triplicate (*n* = 3) and all results were expressed as means ± s.d.

The in vitro release studies were carried out to further understand the release behavior of RIS in implantable devices. All groups were performed until all the drug was completely released from the implantable devices or until the study was stopped. Initially, in vitro, release studies were conducted with implantable devices containing different compositions of drug and excipient under the same thickness of films. The aim of this was to determine the influence of drug composition. The first step was to study the release of RIS from the uncoated pellets. As presented in **Figure** **6**, these curves are all similar and release all the drugs in 20 days. When evaluating similarity and difference factors, if the value of F_1_ is between 0 and 15, and the value of F_2_ is between 50 and 100, then the two release profiles are considered similar.^[^ ^30^ ^]^ As listed in **Table** **4**, the results suggest that the release from R50P50 and R60P40 can be considered equivalent. RIS released from R40P60 and R60P40 cannot be considered equivalent according to F_1_ and F_2_ results. Finally, the results obtained for the comparison of R40P60 and R50P50 curves showed some discrepancies between F_1_ and F_2_ factors but considering how close the calculated values were to the threshold limit it can be assumed that the curves are equivalent. The comparison of F_1_ and F_2_ factors between R40P60 in 4 coatings and R50P50 in 4 coatings, R40P60 in 4 coatings and R60P40 in 4 coatings, and R50P50 in 4 coatings and R60P40 in 4 coatings can also validate above conclusion. Furthermore, by comparing the F_1_ and F_2_ factors between R40P60 and R40P60 in 4 coatings, R50P50 and R50P50 in 4 coatings, and R60P40 and R60P40 in 4 coatings, it can be concluded that they were different in each comparison, which proved the impact of PCL films on slowing release rate.

**Table 4:** Difference and similarity factors calculated of each release profile of implantable devices.

Curve 1	Curve 2	F1	F2
R40P60	R50P50	16	53
R40P60	R60P40	26	43
R50P50	R60P40	11	62
R40P60	R40P60 in 4 coatings	142	18
R50P50	R50P50 in 4 coatings	151	19
R60P40	R60P40 in 4 coatings	63	20
R40P60 in 4 coatings	R50P50 in 4 coatings	17	51
R40P60 in 4 coatings	R60P40 in 4 coatings	26	42
R50P50 in 4 coatings	R60P40 in 4 coatings	9	60
R60P40 in 4 coatings	R60P40 in 5 coatings	23	38
R60P40 in 4 coatings	R60P40 in 6 coatings	54	21
R60P40 in 4 coatings	R60P40 in 7 coatings	58	20
R60P40 in 5 coatings	R60P40 in 6 coatings	41	33
R60P40 in 5 coatings	R60P40 in 7 coatings	47	30
R60P40 in 6 coatings	R60P40 in 7 coatings	12	67
R60C40 in 4 coatings	R60C40 in 5 coatings	36	37
R60C40 in 4 coatings	R60C40 in 6 coatings	48	31
R60C40 in 4 coatings	R60C40 in 7 coatings	48	31
R60C40 in 5 coatings	R60C40 in 6 coatings	17	63
R60C40 in 5 coatings	R60C40 in 7 coatings	19	60
R60C40 in 6 coatings	R60C40 in 7 coatings	3	90
R60P40 in 4 coatings	R60C40 in 4 coatings	29	32
R60P40 in 5 coatings	R60C40 in 5 coatings	42	33
R60P40 in 6 coatings	R60C40 in 6 coatings	19	60
R60P40 in 7 coatings	R60C40 in 7 coatings	21	63

When the pellets were coated with the tubular rate‐controlling membrane, the RIS release rate was slowed down significantly as presented in Figure 6. It is important to note that these results suggest that the use of PCL‐based membranes can sustain the release of RIS for up to 70 days. The release duration is proportional to the drug loading. Lower drug loadings resulted in a shorter release duration (≈40 days). When comparing the results of the RIS release from the pellets with the release from the coated implants F_1_ and F_2_ factors indicate that the release curves are different. The differences between the coated implants are more obvious and it can be established that the higher the drug loading the slower is the release. The comparison of the curves using F_1_ and F_2_ confirms these results. This may be because PEG is a hydrophilic polymer that could assist in the solubilization of hydrophobic drugs. Similar results have been observed previously for other hydrophobic drugs such as olanzapine, where PEG increased the solubility of the drug.^[^ ^28b^ ^]^

Drug release from reservoir systems is influenced by various parameters and typically follows a diffusion‐controlled mechanism.^[^ ^31^ ^]^ For the drug to be released, water must permeate the membrane, partially dissolving the drug within it. The dissolved drug then diffuses through the membrane and is released, creating a concentration gradient between the implant core and its external environment.^[^ ^31^ ^]^ Also, it is important to mention that the concentration gradient and, the release rate, will be proportional to drug solubility inside the implant core.^[^ ^31^ ^]^ If the concentration gradient remains constant, the system exhibits zero‐order release. To sustain this gradient, the core must contain a sufficient quantity of drug to maintain a saturated drug solution. This includes both dissolved drug and undissolved drug reserves. As drug molecules are released, the undissolved drug replenishes the dissolved portion, preserving saturation conditions within the device. However, as the drug load diminishes over time, the system eventually fails to maintain saturation, causing the release rate to slow. This depletion explains the deviation from linearity observed in Figure 6 during the later stages of release.

**Figure 7:**
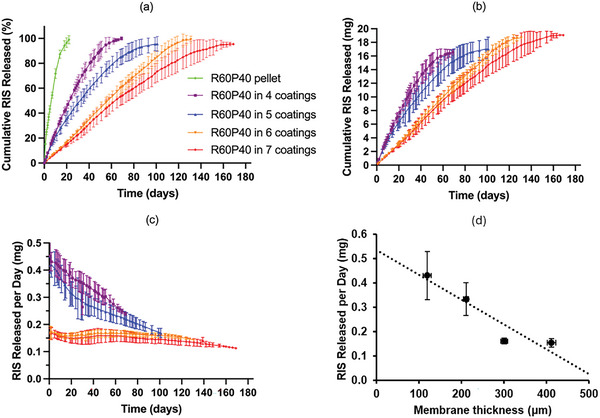
In vitro release profile of R60P40 pellet in different thicknesses of PCL tubes. a) Cumulative RIS released in percentage. b) Cumulative RIS released in milligrams. c) RIS released in mg per day. Release studies were carried out in triplicate (*n* = 3) and all results were expressed as means ± s.d. d) Correlation between membrane thickness and RIS release rate.

**Figure 8:**
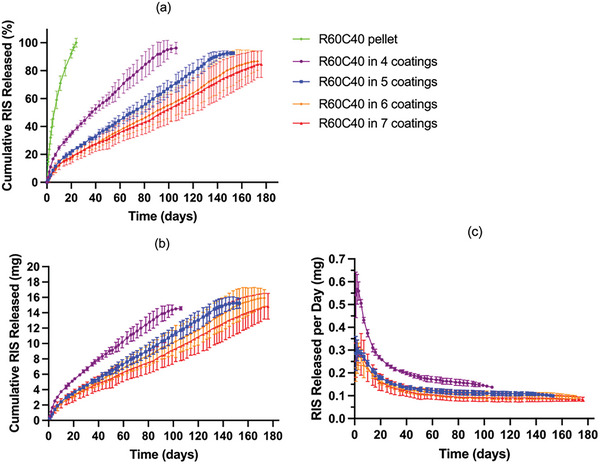
In vitro release profile of R60C40 pellet in different thicknesses of PCL tubes. a) Cumulative RIS released in percentage. b) Cumulative RIS released in milligrams. c) RIS released in mg per day. Release studies were carried out in triplicate (*n* = 3) and all results were expressed as means ± s.d.

Another in vitro release study was performed with implantable devices using directly compressed pellets with the same formulation but coated with different layers of film to evaluate the impact of film thickness. As shown in **Figures** **7**a and **8**a, the release of both RIS‐PEG pellets and RIS‐HPβCD pellets occurred rapidly, in 22 and 24 days, respectively. They cannot be compared with RIS pellets because pure RIS could not form a directly compressed pellet on its own. In Figure 7., all these devices showed sustained release of drugs, and no burst release was observed initially. The results indicated that the release behavior was significantly influenced by the thickness of the films. With an increase in film thickness, the release rate became slower, ranging from 70 to 170 days depending on the thickness of the film. The release rate correlates with membrane thickness in a linear way as shown in Figure 6d. These implantable devices exhibited a linear drug release rate during their release time. Thicker membranes will lead to a longer diffusion path for the dissolved drug. This leads to slower release kinetics as demonstrated previously.^[^ ^26^, ^32^ ^]^

Figure 8 shows the cumulative release profile of implantable devices made from R60C40 pellets and different layers of films. All four groups showed faster release in the first 10 days, after which the release rate became slower, exhibiting a linear drug release rate until all the drugs were released from the implantable devices. The time ranged from 100 to 180 days depending on the thickness of the film. The results suggested that the use of HP‐βCD can be used to extend RIS release. However, as mentioned earlier, these curves showed a biphasic release pattern. The initial release rate is faster due to the presence of HP‐βCD in the pellet. Cyclodextrins and their derivatives have been extensively used to form complexes with drugs and other molecules.^[^ ^33^ ^]^ This excipient increases the solubility of RIS by forming an inclusion complex leading to faster release rates.^[^ ^34^ ^]^ As mentioned earlier, high drug solubility within the implant core leads to faster release rates.^[^ ^31^ ^]^ However, HP‐βCD presents high water solubility and therefore it will be released faster than RIS. Depletion of this excipient will result in a slower release rate as RIS solubility inside the membrane will decrease without HP‐βCD. This is behavior is not ideal as the devices are expected to provide a consistent daily dose of RIS to manage schizophrenia. However, this type of system might be useful for other applications that require a large initial dose and a lower dose at later stages.

To evaluate the release behavior of RIS, the zero‐order release model and first‐order release model were fitted to 60% of the release profile, and the results are shown in **Table** **5**. The kinetic model with an *R* ^2^ value closer to 1 was considered the best fit. It is evident that most of the implants showed a good fit to the First‐order kinetics illustrating that the release profile is concentration‐dependent. However, R60P40 formulations coated with 6 and 7 layers of porous PCL showed a better fit to the zero‐order kinetic model. This indicates that the release profile is independent of the concentration and that the membrane is controlling the release profile rather than the drug concentration gradient. This phenomenon is not observed for equivalent coatings when the solubility enhancer selected was HP‐βCD as described previously.

**Table 5:** The release kinetic analysis of the release behavior of RIS from implantable devices.

	Zero‐order		First‐order	
	*K*_0_ [day^−1^]	*R* ^2^	*K*_1_ [day^−1^]	*R* ^2^
**R60P40 in 4 coatings film**	0.022	0.978	0.030	0.982
**R60P40 in 5 coatings film**	0.016	0.922	0.022	0.956
**R60P40 in 6 coatings film**	0.009	0.986	0.011	0.960
**R60P40 in 7 coatings film**	0.008	0.954	0.010	0.936
**R60C40 in 4 coatings film**	0.014	0.844	0.020	0.944
**R60C40 in 5 coatings film**	0.007	0.914	0.010	0.961
**R60C40 in 6 coatings film**	0.006	0.881	0.008	0.919
**R60C40 in 7 coatings film**	0.006	0.820	0.008	0.862

The results presented here suggest that the resulting implants could provide sustained release over prolonged periods of time of up to 200 days. Previous studies described matrix‐type implants for RIS release showing lower release durations (up to 100 days).^[^ ^35^ ^]^ Also, these systems were not capable of providing linear RIS release. This is commonly observed for matrix‐type implants.^[^ ^36^ ^]^ The literature describes alternative tubular membrane implants for sustained drug release. Those implants could provide sustained release of hydrophobic drugs such as levonorgestrel, etonogestrel, or cabotegravir.^[^ ^19^, ^24^ ^]^ The doses of these compounds are lower than RIS and in that case, implants can be designed for releases of up to 1 year. Interestingly, Li et al. used a similar strategy than the one described here as PCL‐based membranes were used.^[^ ^24^ ^]^ They were prepared via‐hot‐melt extrusion. These implants, like the ones described here, are biodegradable and will not require to be retrieved. Interestingly, the biodegradation of the material described here was evaluated previously showing that the release rate over periods of more than 200 days is not affected by membrane degradation as PCL shows a slow biodegradation rate.

**Figure 9:**
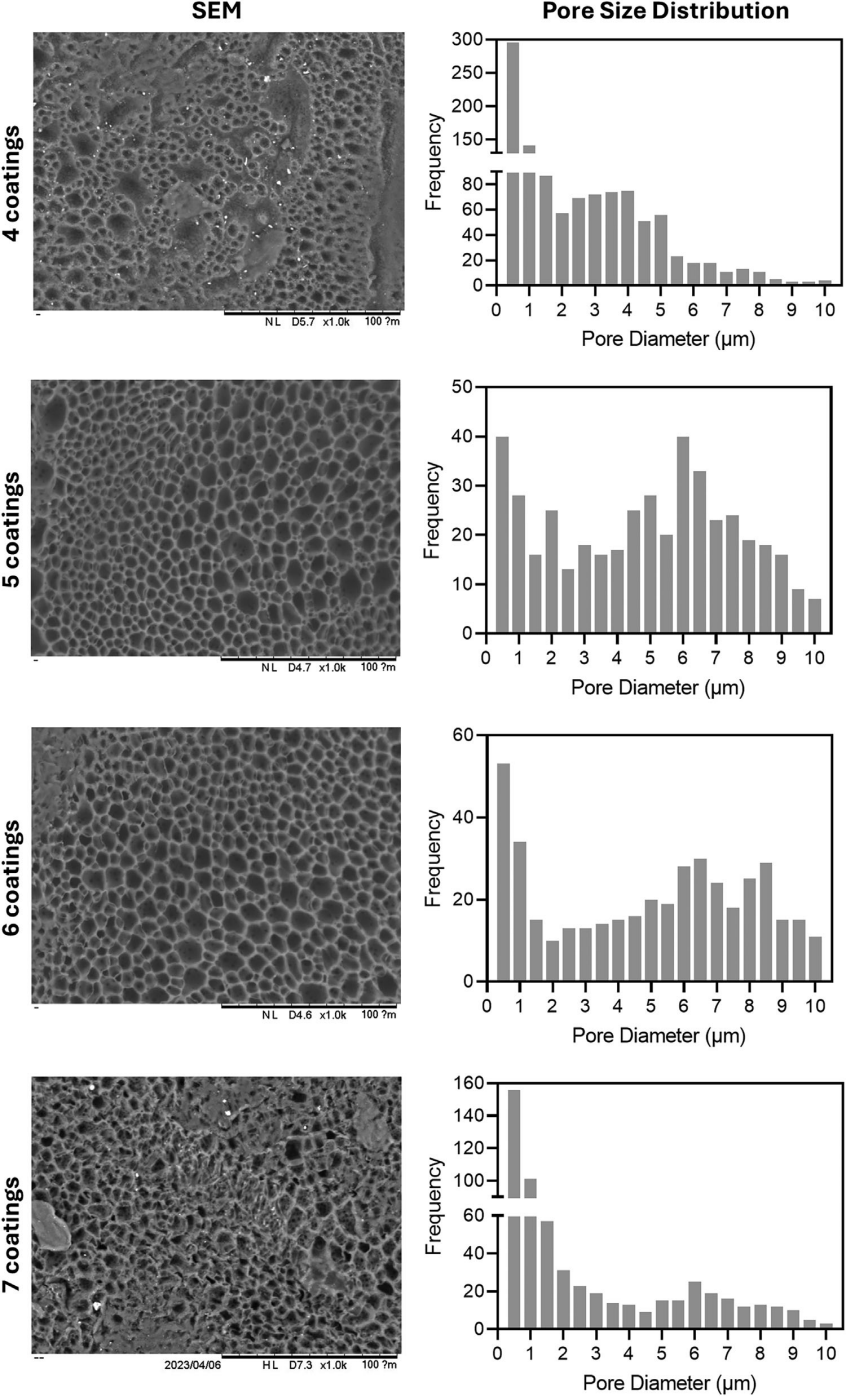
SEM images of the membranes and pore size distribution obtained from SEM images after the release study. Scale bar: 100 µm.

The morphology of films after the in vitro release studies can be observed using SEM and the results are shown in **Figure** **9**. The morphology of the pores is like the morphology of the pores before the release experiment. Additionally, the pore size distribution was measured, and the profiles are like the ones obtained pre‐release experiments. This is consistent with the slow degradation rate of PCL films reported previously. Finally, it is important to mention that other studies showing tubular membranes for sustained drug release were prepared using non‐biodegradable polymers such as poly(urethane) or poly(ethylene*‐co*‐vinyl acetate).^[^ ^19^ ^]^ These implants will need to be removed once they have completed the release of their cargo. Matching the degradation of the implants with the end of the release rate is challenging, and if implant removal is to be avoided, subsequent implants may need to be placed in a different location, allowing time for the leftover polymer from the original implant to fully degrade.

### 2.4 Biocompatibility Studies of RIS Implantable Devices

To figure out the in vitro cytotoxicity and potential applicability of a biomaterial or formulations, the cell viability and proliferation were conducted using HDFa cells. Fibroblasts are formed from mesenchymal tissue and located in the dermis.^[^ ^37^ ^]^ Their primary function is to continuously secrete macromolecules from the extracellular matrix, such as collagen, glycosamin glycanes, and glycoproteins, in order to maintain structural integrity. Damage to tissue causes fibroblasts to proliferate and release cytokines.^[^ ^38^ ^]^ It is generally accepted that using cells homologous to the relevant human tissue will result in more compelling in vitro toxicity assessments.^[^ ^39^ ^]^ Thus, human dermal fibroblasts would be suitable cell lines for assessing local skin compatibility.

**Figure 10:**
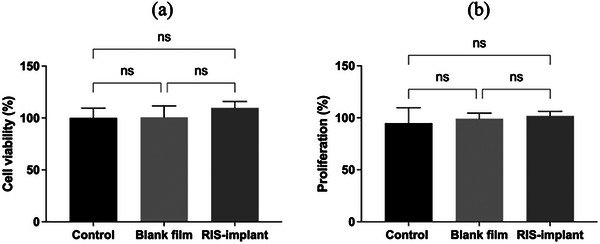
Cytotoxicity Study. a) MTT assay at 48 h, leaching solutions of the formulations and positive control were exposed to cells with media (Means ± s.d., *n* = 5). b) Percentage of DNA concentration shown by the PicoGreen assay (Means ± s.d., *n* = 6).

First, the using 3‐(4,5‐dimethylthiazol‐2‐yl)2,5‐diphenyltetrazolium bromide (MTT) assay was used to detect the influence of formulations on cell viability. It was observed in **Figure** **10**a that after 48 hours incubation, the percentage of cell viability of blank PCL films and RIS‐implantable devices were 100.68% and 109.81%, respectively. Compare to the control group which represented 100% of viable cells, there were no significant difference in blank PCL films (*p* = 0.9907) and RIS‐implantable devices (*p* = 0.1807). Additionally, the presence of RIS pellet in the PCL films also showed no significant difference compared to the blank PCL films (*p* = 0.2223). The results indicate the effect of the formulations on skin cells, have a grade 0 of toxicity (non‐cytotoxic), according to the cytotoxicity grade levels described before.^[^ ^40^ ^]^

To illustrate the cell numbers in the well plates, a PicoGreen assay was carried out to test the total amount of DNA concentration in the medium, which is a good indicator of cell numbers and cell proliferation. It is shown in Figure 10b. Compared to the control group, the samples showed a non‐significant reduction on cell proliferation of HDFa after treatment of blank PCL films (*p* = 0.7290) and RIS‐implantable devices (*p* = 0.4329). The findings align with the viability results. Taken together, it was demonstrated that the formulations have good cytocompatibility, making them promising materials to be used on the skin.

### 2.5 In Vivo Delivery of Risperidone from Implants

**Figure 11:**
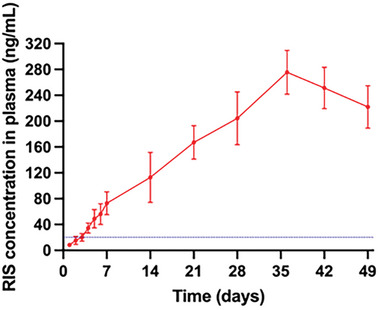
RIS plasma profiles of the rats from the implant cohort following the in vivo study for 49 days. The dot line represents the human therapeutic level of RIS. (Mean ± s.d., *n* = 6).

In this study, male Wistar rats were used as the animal model, and they were applied with implantable devices made from R60P40 pellets with six layers of coatings to investigate the in vivo delivery of RIS from the implantable devices. The pharmacokinetic profiles of RIS are presented in **Figure** **11**., and some relevant pharmacokinetic parameters can be concluded from the figure. The plasma levels of RIS started to increase significantly after the insertion of implants at a relatively constant rate. The human therapeutic level (20–60 ng mL^−1^) has reached at the third day after implantation.^[^ ^41^ ^]^ At 36 days (Tmax) after implantation, the plasma level reached the peak at 275.70 ng mL^−1^ (Cmax). Obviously, this is a pilot experiment to determine if the implants developed in this work were capable of providing sustained drug release. The size and weight of the animal compared with the size of the implant should be considered too. However, in order to evaluate daily release rates, at the end of the experiment, the implants were taken and dissolved in methanol. There were 3.56 ± 0.86 mg of RIS left in each implant, indicating that ≈80% of the RIS has been released from the implantable device. Therefore, and considering that this formulation seems to follow a zero‐order release kinetics it can be concluded that the in vivo release rate is 306 ± 17 µg day^−1^. This release rate is faster than the one obtained during in vivo release. This might be due to the clearing rate of RIS during the in vivo experiments. Additionally, if those release rates could be extrapolated to humans, between 2 and 3 implants will be required to achieve a conventional therapeutic dose of 700 µg day^−1^ (1 mg day^−1^ orally; 70% bioavailability).^[^ ^42^ ^]^ The length of the implants described here (Figure 5) allows to pack 2 or even 3 pellets inside a longer implant. This device will still be within the dimensions of commercially available implants that can be between 4 and 5 cm long.^[^ ^1^ ^]^

This pilot data demonstrates the feasibility of this novel implantable device for RIS delivery. The sustained release over 49 days highlights the potential of this device. Other long‐acting drug delivery systems for the treatment of schizophrenia have been developed. Commercial RIS formulations include in situ forming implants based on PLGA (Perseris) and PLGA microparticles (Risperdal Consta).^[^ ^43^ ^]^ The pharmacokinetic performance of these formulations differs significantly. PLGA in situ forming implants (Perseris) provide a faster drug release, with peak plasma levels observed between 10 and 20 days, followed by a gradual decline lasting ≈70 days.^[^ ^43a^ ^]^ The initial drug peaks are due to an initial fast RIS release reported for this type of formulation followed by a zero‐order release phase.^[^ ^44^ ^]^ In contrast, RIS microparticles (Risperdal Consta) exhibit a lag phase characterized by relatively low drug levels, followed by a delayed peak (30–40 days post‐injection).^[^ ^45^ ^]^ These results are explained by the biphasic release exhibited by RIS‐loaded PLGA microparticles.^[^ ^46^ ^]^ During this lag phase, patients are required to take oral RIS to maintain adequate plasma levels.^[^ ^43a^ ^]^ While the results of these commercial formulations have been obtained in human studies, the pharmacokinetic data presented here are based on rat models. Although a direct comparison of drug levels between species is not possible, the pharmacokinetic profiles can still be compared. The porous membranes used in this study demonstrated a steady drug release without initial fast release, avoiding lag phase or initial early drug level peaks, potentially addressing some limitations of existing systems.

Interestingly, the in vivo release profile of the implants in this study closely resembles that reported by Ibrahim et al. for injectable in situ gel‐forming implants based on PLGA (similar to Perseris).^[^ ^47^ ^]^ Their work evaluated in vivo drug release using a rat model like the one described here. In that study, RIS concentrations increased steadily following administration, reaching a maximum at ≈20 days, and then gradually declined over the subsequent 80 days. It is notable that the reported Cmax for the injectable PLGA implants was ≈160 ng mL^−1^—around half the value observed in the present study. However, it is important to note that the administered dose in their work was over three times smaller (25 mg k^−1^ g) compared to the dose used here (87 mg k^−1^ g, based on an average implant loading of 18.5 mg and an average rat weight of 213 g).

The proposed membranes effectively sustain drug release and have previously been shown to be biocompatible. However, in vivo biodegradation studies should be completed in the future. While PCL is a biodegradable polymer, it degrades more slowly than other biodegradable polymers, such as PLGA. In this case, slower degradation is advantageous as the membrane must maintain its integrity throughout the degradation process. Previous studies have shown that the degradation rate of this polymer is slower than the drug release kinetics.^[^ ^27^ ^]^ The implication is that patients requiring recurrent implants may need the implant to be removed after drug cargo depletion or a new implant to be placed in a different location to allow for full membrane degradation.

## 3 Conclusion

The work described involved the fabrication of the RIS direct compressed technique using two different excipients and PCL films made from high molecular weight PCL and low molecular weight PCL. These implantable devices demonstrated significant potential for enabling sustained release of RIS, as evidenced by a series of characterizations assessing pellet morphology, solidity, drug distribution, and film morphology and thickness. The results indicated that all implantable devices were well‐formed with a uniform distribution of RIS. In vitro release profiles were thoroughly evaluated, revealing both similarities and differences in release kinetics among the devices. By varying the thickness of the PCL films or the type of excipients used in the pellets, the implantable devices exhibited sustained release profiles ranging from 70 to 170 days, characterized by linear release kinetics. Furthermore, preliminary therapeutic efficacy was assessed using an animal model in vivo, demonstrating the feasibility of the study. Future studies should consider extending the duration of release studies, analyzing plasma concentrations of metabolites, and conducting comprehensive investigations into safety, sterility, and stability prior to clinical application. These steps are essential for further advancing the development of these implantable devices for long‐term drug delivery.

## 4 Experimental Section

### Materials

RIS was purchased from Enke Pharma‐Tech Co., Ltd., (Cangzhou, China). PCL Capa 6506 (*Mw* = 50 000 Da), referred to as H‐PCL (high molecular weight PCL), and PCL Capa 2054 (*Mw* = 550 Da), referred to as L‐PCL (low molecular weight PCL), were provided by Ingevity limited (Warrington, UK). Poly(ethylene glycol) (PEG) 8000 (average *Mw* = 8000 Da) was purchased from Sigma‐Aldrich (Dorset, UK). HP‐βCD (Cavitron W7 HP7 PHARMA) was purchased from Ashland (Worcestershire, UK). Moreover, HPLC‐grade acetonitrile, methanol, DCM, and triethylamine were purchased from Sigma Aldrich, (Dorset, UK). Additionally, Phosphoric acid (purity 85%) was purchased from Fluorochem Limited (Hadfield, UK) and phosphate‐buffered saline (PBS) tablets (pH 7.4) were purchased from Sigma‐Aldrich (Dorset, UK). Sodium azide was purchased from Fluorochem Limited (Hadfield, UK) and all other chemicals used were of analytical reagent grade. Finally, deionized water was obtained from ELGA purified, PureLab water purification system (High Wycombe, UK).

### Fabrication of Directly Compressed RIS Pellets

Directly compressed pellets containing RIS were prepared using a Specac Atlas manual hydraulic press (Specac Ltd, Kent, UK) using a 3 mm diameter punch and die system. Each formulation, as detailed in **Table** **6**, was mixed using a pestle and mortar. Then, a certain quantity of the formulation was weighed accurately and transferred to the manual hydraulic press. A pressure of 1 ton was applied for 20 s, and then the pellet was removed from the apparatus. After the fabrication of all pellets was completed, they were weighed again, and the actual quantity was recorded, as there might be some loss during the manufacturing process.

**Table 6:** Formulations containing RIS for the fabrication of directly compressed pellets.

Formulation	RIS % [w/w]	Excipient Type	Excipient % [w/w]
R40P60	40	PEG 8000	60
R50P50	50	PEG 8000	50
R60P40	60	PEG 8000	40
R75P25	75	PEG 8000	25
R100	100	–	–
R60C40	60	HPβCD	40

### Determination of Hardness of RIS Pellets

To investigate the maximum force required to fracture the RIS pellet, the Pharmatron tablet hardness tester (Copley Scientific, Nottingham, UK) was used. The pellet was placed on the stage, and the stainless‐steel compressor began by measuring the diameter of the pellet. Then, the stainless‐steel compressor moved slowly toward the pellet until it made contact, and compression continued until the pellet fractured. The formulation process was repeated five times (*n* = 5).

### Fabrication of PCL Films and Implant Preparation

In this study, the pellet was wrapped with a PCL film. According to previously published studies, the PCL films were fabricated using high molecular weight PCL (*Mw* = 50 000 Da) and low molecular weight PCL (*Mw* = 550 Da) with a ratio of 2:3.^[^ ^27^ ^]^ The mixture of PCL was dissolved in DCM and used to fabricate the film with an overhead stirrer IKA RW 20.n (IKA Works Inc., Wilmington, NC, USA) and stainless‐steel rod. The overhead stirrer was placed in a fume hood to evaporate the solvent. A 15 mL Falcon tube was used as the container for the PCL solution to allow the rod to dip in and out. The rotation speed was set to 60 rpm, and each layer was rotated for 10 min to evaporate the DCM. Central sections of the tubular membrane were used to assemble implants to keep membrane thickness consistent. Moreover, a single implant was prepared from each tubular membrane prepared.

The RIS implantable device was assembled using a PCL tube and directly compressed pellets containing RIS. The pellet fitted directly into the tube because the rods used to compress pellets and fabricate films were the same size. A pair of pliers was heated in the oven at 80 °C for 1 min and then used to seal both ends of the film.

### Optical Coherence Tomography Analysis

To obtain cross‐sectional images of the films and implantable devices, assess the thickness of the films, and visualize the pellet‐filled implant, an EX1301 OCT microscope (Michelson Diagnostics Ltd., Kent, UK) was used. The films and implants were placed on the stage, and then the height was adjusted to obtain clear images.

### Mercury Intrusion Porosimetry (MIP)

To further investigate the morphology of the polymeric membranes, after performing the SEM analysis, their pore size distributions were measured by using a Quantachrome PoreMaster 60GT mercury porosimeter (Quantachrome Instruments, FL, USA) using membrane samples of 4, 5, 6, and 7 cycles. The area of the membranes was 1 cm × 9.20 ± 0.11 mm and the thickness ranged from 129.99 ± 9.98 µm to 446.50 ± 11.33 µm depending on the number of the applied cycles in each sample. All samples were placed into a penetrometer to ensure the correct mercury fill into the voids. The relationship between applied pressure and pore size was defined by the Washburn equation, which assumed a relationship between the applied pressure and pore diameter using the physical properties of a non‐wetting material (in this case, mercury which has a contact angle of 140° with the test materials). The applied pressure ranged from 1 to 40 000 psi.

### In Vitro Release Studies

An in vitro release study was conducted to evaluate the release profile of RIS from the implant. Each implant was placed in 200 mL PBS (pH 7.4) to ensure the sink conditions. Meanwhile, 0.2 mL of sodium azide was added to prevent the growth of microorganisms in the release medium. Finally, the sealed containers were placed in an incubated shaker at 40 rpm and 37 °C. One milliliter of the medium was taken at predetermined time intervals and replaced with an equal volume of fresh PBS solution. Then, the samples were centrifuged and analyzed to quantify the RIS in the medium using the validated HPLC method described in the following section. The samples might be diluted to a concentration within the range of the linear calibration curve when needed. Each formulation was prepared and analyzed in triplicate (*n* = 3).

The percentage of drugs released could be calculated using Equation (1).

(1) $$\%\;Drug\;released=\frac{x\times f_{d}\times V}{W}\times 100$$

where *x* is the concentration calculated according to peak area in HPLC analysis, *f_d_* is the dilution factor, *V* is the volume of the sample, and *W* is the amount of RIS in the implantable device.

The similarity (F2) and difference (F1) factors of the in vitro release profile of implantable devices were calculated and compared, as several authors have done in the past.^[^ ^27^, ^48^ ^]^ To assess the similarity of the dissolution profiles, at least 12 units of both the test (T) and reference (R) products were needed, according to recommendations from the EMA and FDA.^[^ ^49^ ^]^ The difference factor was the measurement of the percentage difference and relative error between two curves at each time point, and it could be calculated using Equation (2).

(2) $$F_{1}=\left\{\left\{\left[\sum_{t=1}^{n}\left(R_{t}-T_{t}\right)\right]/\left[\sum_{t=1}^{n}R_{t}\right]\right\}\times 100\right.$$

where *n* is the number of time points, *R_t_* is the reference dissolution value at time *t*, and *T_t_* is the test dissolution value at time *t*.

The similarity factor was a logarithmic sum‐squared error of the difference between the reference and test product over all time and could be calculated using Equation (3).

(3) $$F_{2}=50\;log\left\{{\left[\left(\frac{1}{n}\right)\sum_{t=1}^{n}\left(R_{t}-T_{t}\right)\right]}^{-0.5}\times 100\right\}$$

If the value of F_1_ is between 0 and 15, and the value of F_2_ is between 50 and 100, then the two release profiles are considered similar.^[^ ^30^ ^]^

To further explain the release behavior of different formulations, the data were then evaluated using mathematical kinetic models. In both kinetic models, 60% of the cumulative released profiles were used to fit the models. The kinetic model whose *R* ^2^ value was closest to 1 was considered the best fit.

Zero‐order release kinetics was an ideal behavior for formulation. It was the process by which drugs were released in the same amount throughout time regardless of their concentration in the matrix. Zero‐order release kinetics could be described using Equation (4).

(4) $$Q=Q_{0}+K_{0}\;t$$

where *Q* is the amount of drug release, *Q*_0_ is the amount of drug at start time, which is usually 0, *K*_0_ is the zero‐order release constant, and *t* is time.^[^ ^32a^ ^]^

The first‐order release kinetics described a scenario where the rate of release of the drug was proportional to the concentration of the substance that remained in the formulation. It could be described using Equation (5).

(5) $$\ln Q_{t}=\ln Q_{0}+k_{1}t\;$$

where *Q_t_* is the amount of drug dissolved at time *t*, *Q*_0_ is the initial amount of the drug, *k*_1_ is the first‐order release constant.^[^ ^32a^ ^]^

### Surface Morphology Observation

To observe the morphology of films, a scanning electron microscope (SEM) TM3030 (Hitachi, Krefeld, Germany) was used under a voltage of 15 kv and 1000× magnification. The films were observed before and after the in vitro release study.

### Biocompatibility Studies of RIS Implantable Devices

Cytotoxicity of tested Blank PCL film and RIS implantable devices were tested in vitro using 3‐(4,5‐dimethylthiazol‐2‐yl)2,5‐diphenyltetrazolium bromide (MTT, Sigma Aldrich, St. Louis, MO, USA), on Adult human dermal fibroblasts (HDFa) cells. For testing HDFa cells (density 5 × 103 cells well^−1^) were cultured on 48 well culture plates in DMEM (Sigma‐Aldrich, Saint Louis, USA) containing 10 wt.% fetal bovine serum (FBS) (Sigma‐Aldrich, Saint Louis, USA) and 1 wt.% penicillin‐streptomycin aqueous solution (Sigma‐Aldrich, Saint Louis, USA). MTT assay was used to test cell viability and the test was performed as follows: formulations with cells were treated with MTT reagent at a concentration of 5 mg mL^−1^ after 48 h of culture and incubated for 5 h at 37 °C in a 5% CO_2_ atmosphere.^[^ ^50^ ^]^ The formulations were rinsed with PBS and DMSO was added to dilute the formazan crystals formed. Absorbance at 570 nm was measured using a Multiskan GO spectrophotometer (Thermo Fisher Scientific, USA).

The PicoGreen (DNA quantification) assay was used to quantify the DNA (cell proliferation) content on cultured cells and was performed as follows. The calibration curve was made from the DNA standard for the determination of unknown concentrations of the DNA. The cells with or without formulations were incubated with leaching solution for 48 h, then formulations were rinsed with PBS three times and submerged in 1 mL of lysis buffer containing 10 mm Tris (pH 8), 1 mm EDTA, and 0.2% (v/v) Triton X‐100. To release the DNA, the samples were vortexed for 10 s every 5 min for a total of 30 min and were kept on ice throughout the entire process. The samples were thawed on ice, and homogenized 10–15, after, the sample was mixed with 100 µL of DNA‐binding fluorescent dye solution and incubated at room temperature for 5 to 8 min in the dark. To calculate the DNA concentration in the samples, the fluorescence was measured at 480 nm excitation and 520 nm emission using a Varioskan Flash Thermo Scientific reader plate.

### In Vivo Delivery of Risperidone from Implants

The in vivo pharmacokinetic study of RIS from the implant formulations was approved by the Ethical Committee of Hasanuddin University, Indonesia (protocol number is 201 022 105 031). Healthy male Wistar rats (average mass of 213 ± 12 g) were adapted to the laboratory environment for 1‐week period prior to the experiment. For this matter, as you suggested, the implants were placed in PBS 4 h in a fume hood equipped with UV light for to ensure the sterility of the formulation before the implantation. Initially, rats were sedated using ether. Following this, the hair from their dorsal was removed with hair removal cream. Subsequently, the hairless area was rubbed with an antiseptic solution and a 20 mm dorsal midline incision was created. Finally, the implants were inserted subcutaneously at the incised sides (1 implant per rat). The average RIS content per implant was: 18.5 ± 1.3 mg. To assess the plasma pharmacokinetics, blood samples were collected at day 1 until day 28 after the implant administration. The blood obtained was collected into an Eppendorf tube containing 3.8% w/v of sodium citrate to prevent blood coagulation. The blood was spun for 10 min at 4 °C at 3000 × g, obtaining the plasma samples. The plasma was stored at −20 °C prior to analysis.

RIS quantification was performed using an HPLC system (Shimadzu Prominence, Shimadzu, Kyoto, Japan) equipped with an Xselect CSH C18 column (Waters, 3.0 × 150 mm) with a particle size of 3.5 µm. The mobile phase was a mixture of 10 mm sodium dihydrogen phosphate buffer (with 1% v/v triethylamine, pH 7.2 adjusted using orthophosphoric acid) and a mixture of methanol and acetonitrile (75:25% v/v) with a ratio of 15:85% v/v. The detection was performed using a UV detector at 235 nm, with an injection volume of 20 µL and a flow rate of 0.5 mL min^−1^ at 25 °C. Chromatogram analysis was performed using Shimadzu LC solution software (ver. 1.21 SP1).

### Data Analysis

All results were expressed as means ± standard deviation. Statistical analysis and mathematical kinetic models were carried out using GraphPad Prism version 10.2 (GraphPad Software, San Diego, CA, USA). The *t*‐test was used to analyze results from two cohorts, while one‐way ANOVA was used to analyze results from more than two groups. *P* < 0.05 denoted significance in all cases. ImageJ software (National Institutes of Health, Bethesda, MD, USA) was used to process OCT images.

## Conflict of Interest

The authors declare no conflict of interest.

## Data Availability

The data that support the findings of this study are available from the corresponding author upon reasonable request.
